# Building a decision-support tool to inform sustainability approaches under complexity: Case study on managing wild ruminants

**DOI:** 10.1007/s13280-024-02020-9

**Published:** 2024-04-17

**Authors:** Paul Griesberger, Florian Kunz, Klaus Hackländer, Brady Mattsson

**Affiliations:** Institute of Wildlife Biology and Game Management, Department of Integrative Biology and Biodiversity Research, University of Natural Resources and Life Sciences, Vienna, Gregor-Mendel-Str. 33, 1180 Vienna, Austria

**Keywords:** Bayesian decision network, Collaborative decision analysis, Decision-support tool, Resource allocations, Stakeholder workshops, Wildlife-based conflicts

## Abstract

In wildlife management, differing perspectives among stakeholders generate conflicts about how to achieve disparate sustainability goals that include ecological, economic, and sociocultural dimensions. To mitigate such conflicts, decisions regarding wildlife management must be taken thoughtfully. To our knowledge, there exists no integrative modeling framework to inform these decisions, considering all dimensions of sustainability. We constructed a decision-support tool based on stakeholder workshops and a Bayesian decision network to inform management of wild ruminants in the federal state of Lower Austria. We use collaborative decision analysis to compare resource allocations while accounting for trade-offs among dimensions of sustainability. The tool is designed for application by non-technical users across diverse decision-making contexts with particular sets of wildlife management actions, objectives, and uncertainties. Our tool represents an important step toward developing and evaluating a transparent and replicable approach for mitigating wildlife-based conflicts in Europe and beyond.

**Supplementary Information:**

The online version contains supplementary material available at 10.1007/s13280-024-02020-9.

## Introduction

Governments around the world have declared that managing and conserving wildlife along with associated ecosystem services are important means to achieve the Sustainable Development Goals (SDGs; Secretariat of the Convention on Biological Diversity 2014; UN General Assembly 2015). This is a great challenge, as wildlife species along with their habitats are threatened by anticipated impacts of climate change and increasing demands for natural resources (IPCC 2014). Achieving sustainability should correspond with a thriving ecological system typified by diverse communities of free-living vertebrates (henceforth, wildlife) coexisting with humans, which is also reflected in the Aichi Biodiversity Targets adopted by the Convention on Biological Diversity (CBD Secretariat 2010).

Originally shaped by the Brundtland Report (WCED 1987) and the Agenda 21 of the United Nations Conference on Environment and Development (UNCED 1992), sustainability is nowadays widely acknowledged to be represented by three dimensions, namely Ecology, Economics and Socioculture (Purvis et al. 2019). To guide the sustainable management of natural resources, 17 SDGs were specified at the United Nations Sustainable Development Summit 2015 (UN General Assembly 2015). Wildlife species and associated natural resources are highly valued by people around the world (Schulp et al. 2014; Gren et al. 2018; Subroy et al. 2019) and their management is thus integral in many SDGs. Strategies to manage wildlife, however, have diverse effects on these species, their habitats and people involved in any kind, spanning all three dimensions of sustainability.

Some people directly benefit from wildlife by non-consumptive (observing) or consumptive (hunting) use of these animals (Schulp et al. 2014; Mattsson et al. 2018), while others derive relational or nonuse values (Subroy et al. 2019). Still others perceive wildlife populations as being overabundant and posing threats to their social and economic well-being (Gren et al. 2018). Contrasting perspectives about wildlife management generate conflicts between people that are expressed in the media or public protests (Redpath et al. 2015; Nyhus 2016; Madden and Mcquinn 2017; Pooley et al. 2017), which we refer to henceforth as wildlife-based conflicts or simply as conflicts. Actual and perceived impacts of wildlife on human livelihoods can generate conflicts about how to best manage these species. Opposing stakeholders may refuse to communicate, juxtaposed values preclude easy win–win solutions, and sensational media can exacerbate the differing sides to debates (Redpath et al. 2013). Wildlife-based conflicts therefore can manifest as wicked problems that undermine natural resource management and challenge achievement of the SDGs and Aichi Targets. Further, such conflicts are ill-structured, and therefore difficult to address with unilateral approaches (e.g., law enforcement and damage compensation programs; Marino et al. 2021). Decision-making processes, if well designed, can help to structure and mitigate wildlife-based conflicts (Mattsson et al. 2019). As such, collaborative decision analysis (CDA; Thorne et al. 2015) is a process that integrates methods of stakeholder engagement (Reed 2008), structured decision making (Runge et al. 2022), and multi-criteria decision analysis (Adem Esmail and Geneletti 2018) to inform natural resource management. Owing to the transparent methodology including stakeholder involvement, CDA has been useful for addressing wildlife-based conflicts (e.g., Mitchell et al. 2018; Mattsson et al. 2019; Marino et al. 2021; Johnson et al. 2022). CDA allows for incorporating perspectives of multiple stakeholders with differing viewpoints within the decision-making process, from conceptual framing of the decision problem through quantitative comparison of management options.

Existing applications of CDA to wildlife-based conflicts exhibit at least one of three challenges. First, these efforts have been tailored to specific decision contexts and many lack clear relevance to be applied across cases and regions (e.g., Mustajoki et al. 2011; Mattsson et al. 2019). Second, previous applications involved a combination of decision-analytic tools and expert elicitation that require high levels of expertise to properly conduct (e.g., Mustajoki et al. 2011; Johnson et al. 2022). Third, many studies have only involved up to two dimensions of sustainability (i.e., ecological and sociocultural) and have not integrated the economic dimension (e.g., Marino et al. 2021; Johnson et al. 2022). For this reason, their relevance to the SDGs is limited. To our knowledge, no applications to wildlife management (for other applications see review in Diaz-Balteiro et al. 2017) have addressed all three dimensions at once. An accessible, transferable, and multi-dimensional framework for decision-making is needed to inform sustainable management of wildlife in the face of conflicts.

As a prominent example of wildlife-based conflicts, numbers of wild ruminants are increasing in many human-dominated landscapes across Europe (Apollonio et al. 2010) and beyond (Cromsigt et al. 2013). This increase often leads to negative effects at fine spatial scales, like damages on forests through browsing or bark stripping (Carpio et al. 2021). Elevated levels of herbivory may exceed ecological tipping points and compromise the integrity and functionality of forest functions, causing economic, ecological, and sociocultural degradation (Gerhardt et al. 2013). Conflicts between stakeholders about regulating herbivory, in turn, may hinder effective measures to minimize these negative impacts (Hodgson et al. 2020). Collaborative approaches to support decisions may help mitigate conflicts when managing overabundant populations of wild ruminants. Particularly hunting practices play an important role by regulating population numbers of herbivorous game species through harvesting and modifying spatial distribution of these species to reduce impacts on vegetation (Cromsigt et al. 2013; Heurich et al. 2015). As current hunting practices often fail in this context, however, conflicts triggered by wild ruminants are still a major problem in many regions (Valente et al. 2020). Recognizing these conflicts and balancing sustainability objectives are common challenges in game management (Law et al. 2021). Therefore, science-based methods and tools to inform sustainable management of overabundant wildlife populations are urgently needed in many regions worldwide.

In Austria (Central Europe), the hunting system with its associated laws and culture, provides an overarching framework within which decision makers act to influence ruminant populations and their interactions with the environment. Hunting is strictly bound to real estate, and hunting territories are either individual grounds when they exceed a minimum area of 115 ha or summarized into communal grounds. Landowners are free to hunt themselves or lease the territories (for details on hunting management in Austria see Trouwborst and Hackländer 2018 or Reimoser and Reimoser 2010). Hunting legislation is subdivided by provinces, with each Austrian province having its own hunting law. Regulations under these laws include annual harvesting of game species and economic compensation for game damages on forest vegetation and agricultural crops.

In the province of Lower Austria, hunting territories are leased or hunted for a time span of nine years, while adaptive game harvest plans are prepared by the local authorities for three-year intervals. Hunting in Austria is a long-standing tradition and is a substantial part of rural culture. Within Lower Austria, hunting bags for wild ruminants for the hunting season 2020 totaled 92 545 individuals that were shot by hunters (88% roe deer (*Capreolus capreolus*), 8% red deer (*Cervus elaphus*), 2% chamois (*Rupicapra rupicapra*) and 2% European mouflon (*Ovis gmelini*), fallow deer (*Dama dama*), sika deer (*Cervus nippon*) and Alpine ibex (*Capra ibex*); Statistics Austria 2022). In accordance with a high abundance of wildlife, wildlife-based conflicts are diverse (i.e., economic losses due to impact on vegetation, animal-vehicle crashes with economic and personal damage, tourism activities disturbing hunting activities) and sustainable management of wild ruminants is highly needed.

Our aim is to create a decision-support tool, taking into account the perspectives of diverse stakeholders regarding all three dimensions of sustainability in the management of wild ruminants. The decision context centers on Lower Austria and conflicts related to roe deer, red deer, and chamois. The management of these species involves many decision-making entities who often lack clearly defined objectives or management actions along with uncertainties about management effectiveness (Reimoser and Reimoser 2010). Through the principles of CDA and stakeholder workshops, we elicit and define existing problems, interests and objectives. Further, we determine possible actions to mitigate influencing factors that are at least partly beyond control of decision makers. In the backend of the tool, a Bayesian decision network enables a quantitative comparison of expected utilities between decision options. We base the frontend structure of the tool on perspectives and needs of decision makers and stakeholders, which we gather using focus group discussions and questionnaires. Thus, our tool can be easily applied by practitioners to address complex situations and inform their decisions on resource allocation while maximizing expected satisfaction of stakeholders. Ultimately, our study demonstrates how easy-to-use decision tools for practitioners can be developed to support sustainable management of wildlife in the face of wildlife-based conflicts.

## Materials and methods

### Overview

We used the principles of CDA (Mattsson et al. 2019) and structured decision making (Runge et al. 2022) for designing the decision-support tool along with stakeholder workshops and surveys (see Appendix S1 regarding questions asked during these surveys). In particular we followed the PrOACT steps, including Problem framing, identifying Objectives and Actions, followed by modeling Consequences and Trade-offs between objectives (Mattsson et al. 2019). We supplemented this in two ways. Within the problem-framing step, we identified stakeholders that influence or are affected by the decision. Immediately after identifying objectives, we then specified external factors that can affect the objectives but are at least partly beyond control of the decision maker. These external factors provided important context for developing a resource allocation beyond the status quo within the actions step of the PrOACT process. To ensure the tool was coherent and accessible, we used an iterative and rapid prototyping approach by briefly revisiting individual PrOACT steps and revising the tool when needed. The intended user of our tool was a decision maker responsible for managing wild ruminants in Lower Austria, subsequently termed ‘user’.

Throughout the study and corresponding PrOACT steps, we used several methods of knowledge synthesis (Pullin et al. 2016; Dicks et al. 2017) to extract necessary information for developing the tool. These methodologies included a non-systematic literature review, group discussions within a facilitation team (researchers/analysts), group discussions during six stakeholder workshops, semi-structured interviews, and individual questionnaires for stakeholders. The literature sources included an indicator framework for sustainable hunting in Austria by Forstner et al. (2006) and an extension of this framework (Daim et al. 2017). Online questionnaires were administered approximately two weeks before the second through fifth workshop. Anonymous responses from the questionnaires were summarized, presented, and discussed during the workshops, giving participants the opportunity to clarify or reflect on their responses.

The facilitation team used a consistent process for identifying the list of possible stakeholders, objectives, external factors, and actions (henceforth, factors). Redundant factors were combined, and others were eliminated if they fell outside the decision context. When factors were identified, a concise phrase and definition was constructed for each factor to ensure they were unique and would be understandable for practitioners. The facilitators presented the proposed phrases to the workshop participants during multiple workshops, individual questionnaires, and via email. The facilitation team then adapted the objectives based on feedback from participants. This approach ensured that the factors available for selection in the decision tool were relevant for real-world decisions. In the following subsections we provide justifications and details about our preparation, communication, and tailoring of the PrOACT steps for developing the decision-support tool.

### Preparatory phase and communication throughout the study

Prior to the development of the decision tool, a preparatory phase included the formation of a facilitation team consisting of three researchers with expertise in decision analysis, workshop facilitation and the ecology and management of wildlife in Austria. The core team included the facilitation team and the deputy director of the forestry agency in Lower Austria. This decision maker was included in the core team, as the decision context involved mitigating the impacts of wild ruminants on forest vegetation. During an initial one-hour meeting, roles of the core team members were discussed. In particular, it was determined that the facilitation team should play a neutral role by leading the facilitation of workshops and further assist the decision-making process.

The facilitation team met in person and over video conference every one to three weeks throughout the study. These meetings helped to ensure that the tool provided a robust decision analysis while remaining accessible for workshop participants. This team asked themselves questions such as, “Will a decision maker know what information to enter in the tool?” or “Does this set of entries in the tool provide a correct comparison of decision options?”. Further, the facilitation team led a series of stakeholder workshops. Regarding the decision context, the first three workshops focused on the province level of Lower Austria. Stakeholders from the district level were invited as part of the last three workshops to ensure that this local level is also incorporated within the decision-making process. The workshops were held at intervals of two to four months and lasted four to seven hours each.

During these workshops the facilitators presented the aims of the study along with the current structure of the tool, facilitated open discussions, and administered individual questionnaires to elicit necessary information for developing the tool. Facilitators asked questions including, “Are all potentially relevant objectives available for selection?”. Furthermore, three semi-structured interviews in the form of video conferences with the provincial authority were held between individual workshops to discuss the progress of the tool. The facilitators used these opportunities to ensure that each new version of the tool met the needs and expectations of the authority. This provided an important series of checks before presenting these versions to all participants. Questionnaires outside of workshops were additionally used to get further feedback from the participants regarding the progress of the tool.

### Stakeholder selection and decision question

During an initial conversation, the core team confirmed that the forestry agency representative would have the authority to implement possible recommendations from the CDA process. Consequently, the forestry agency represented a key decision maker and likely user of the decision tool. The core team began by identifying stakeholders that should be considered regarding the management of wild ruminants within but not limited to Lower Austria. Stakeholders were selected based on assessments and prior knowledge of the core team. In particular, the core team identified organizations who are responsible for managing wild ruminants and their habitats. Next, representatives of each stakeholder group were identified for participation in the decision-analytic process. The core team selected individuals who had practical experience with the management of wild ruminants in Lower Austria and are able to make management decisions at the province or district level. As coordinating a large group of participants through workshops would require professional facilitation, the total number of stakeholder representatives was limited to ten.

During the first two stakeholder workshops, the main goal was to describe the current status and perspectives regarding the management of wild ruminants and to identify existing problems in this context. Subsequently, defined problems were checked with the participants whether they were within their authority to address through decision-making. An overarching decision question was formed based on these problems. This decision question included a brief description of the decision maker, the overarching objective, and the spatial extent. During these initial workshops the facilitators clarified with participants the spatial resolutions, temporal scales, and legal framework for the focal decisions. Within the framework of workshops three to six, participants were asked to define suitable management objectives, external factors, and actions, with regard to the decision question. Further, these workshops were used by the facilitators to present prototypes of the tool, which were continuously adapted based on the feedback of the participants.

### Factors

To define management objectives for inclusion in the tool, each workshop participant independently identified concerns and wishes from their perspective as a stakeholder. The facilitators elicited these concerns and wishes during the first workshop by having the participants write them down on sheets of paper and then displaying them on the wall. Between workshops the facilitators converted these concerns and wishes into objectives using the verb–noun format (e.g., maintain abundance of red deer) and presented them to participants during subsequent workshops to ensure the meaning was captured. The facilitators assigned each objective to one of the three dimensions of sustainability, resulting in ecological, economic, and sociocultural objectives. External factors were defined as factors having strong potential influence on objectives and are mostly beyond the direct control of decision makers. These were important to consider before identifying actions, as the actions and associated decision options should aim to address the influence of these external factors when trying to achieve the objectives. When developing an interface within the tool to elicit decision options, the facilitation team considered the maximum number of actions and allowed the user to choose between types of resources to be invested in each action. This was important to ensure that the tool remained simple to use while allowing for a realistic set of actions to compare.

### Consequences and trade-offs

Based on discussions during the workshops and responses to the surveys, the facilitation team assessed the importance of accounting for uncertainties when predicting effects of decision options and external factors on the objectives. This allowed for choosing an appropriate framework for the decision analysis underlying the tool. The facilitation team also considered ways of reducing the level of complexity of the predictive models and the commensurate elicitation burden. Simplifications were done judiciously to ensure that the tool could inform real-world decisions. These approaches included limiting the number of objectives, external factors, and actions that users could select. Another approach to reduce the elicitation burden was indirectly acquiring numerical inputs by asking the user to respond to discrete-choice questions.

As the aim of the study was to develop a decision-support tool to inform management of wild ruminants by accounting for the three dimensions of sustainability, the tool requires that the user specifies multiple objectives. To minimize complexity of the tool despite having multiple objectives while accounting for uncertainty, the facilitation team considered two alternatives for eliciting trade-offs between objectives. First, the relatively time-intensive approach of directly eliciting utility values for every combination of outcomes (Cinelli et al. 2014). Second, an approach that minimized elicitation burden by asking the level of importance for each objective and transforming these entries into utility values in the backend of the tool.

## Results

### Overview

The end product of this study was a decision-support tool that was implemented using Microsoft Excel 2016 and the associated Visual Basic Editor for macros (Appendix S2). Responses to surveys and workshop discussions informed the structure and content of the tool. In short, workshop participants wanted the decision-support tool to be intuitive and customized for their decision context. They wanted a tool that they could use on their own rather than rely on a facilitator using software, especially designed for decision analysis (e.g., Netica; Norsys 2016). Thus, the facilitators used Excel for implementing the tool, as this software is widely applied and familiar to many users. Excel does not require extensive training for basic functionalities and is equipped with many built-in functions that simplify complex calculations. As such, Excel offered the facilitators the necessary features to implement the decision-support tool rapidly and minimized time between rounds of feedback on the tool design. Excel files are easily shared and edited, as this software is not only available for Windows but also for macOS and mobile devises (iOS and Android). To simplify the applicability of the tool in Lower Austria, we developed a user manual (in German) with step-by-step instructions on applying the tool. The tool was named JAKEtool based on the project acronym that refers to the project title in German.

**Fig. 1:**
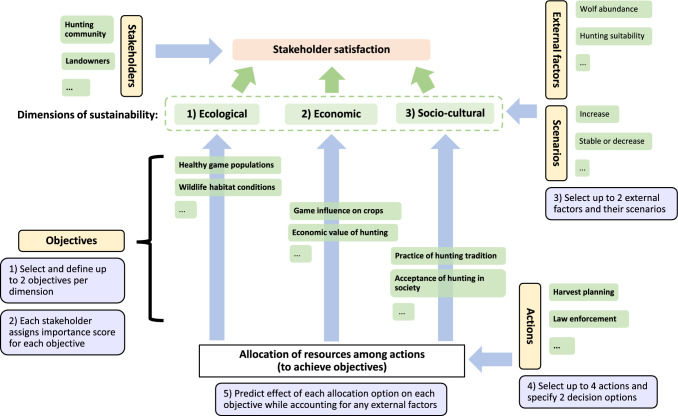
Influence diagram illustrating the structure of the JAKEtool, including the sequence of elicitation steps (white boxes) and example selections from the factor catalog (green boxes) for each factor (yellow boxes). Blue boxes: steps of using the tool

The JAKEtool includes a frontend for users to provide information, some of which was for their own reference, while other entries are used for computations in the backend that are invisible to the user. The tool is embedded in an Excel workbook that contains a number of worksheets. The structure of the tool is shown in Fig. 1. The layout of some worksheets depends on the entries in previous sheets. Therefore, it is important to enter the information starting with the first worksheet. The user fills the tool with information starting with worksheet 1 and working through the other worksheets from left to right. The user can be a single decision maker or a facilitator who gathers the necessary information. It is recommended that the first use of the tool be guided by a facilitator.

Given that decisions are made for individual management units with particular stakeholders, objectives, external factors, and actions, the tool allows the user to select such factors from a list (henceforth, factor catalog; Table S1). To ensure the result is relevant for their management context, proposed definitions may be adapted by the user. Such definitions of objectives, actions, and external factors must be clear and sufficiently detailed to assure practical implementation and traceability regarding the logic of these factors. This helps to guarantee that future users applying the tool for a given management unit can understand and build on earlier applications of the tool. The number of factors that can be selected by the user was limited to ensure the usability of the tool while ensuring enough complexity to inform real-world management of wild ruminants.

In the following sections, we describe how the results of each PrOACT step informed the structure and content of the JAKEtool.

### Stakeholder selection and decision question

**Fig. 2:**
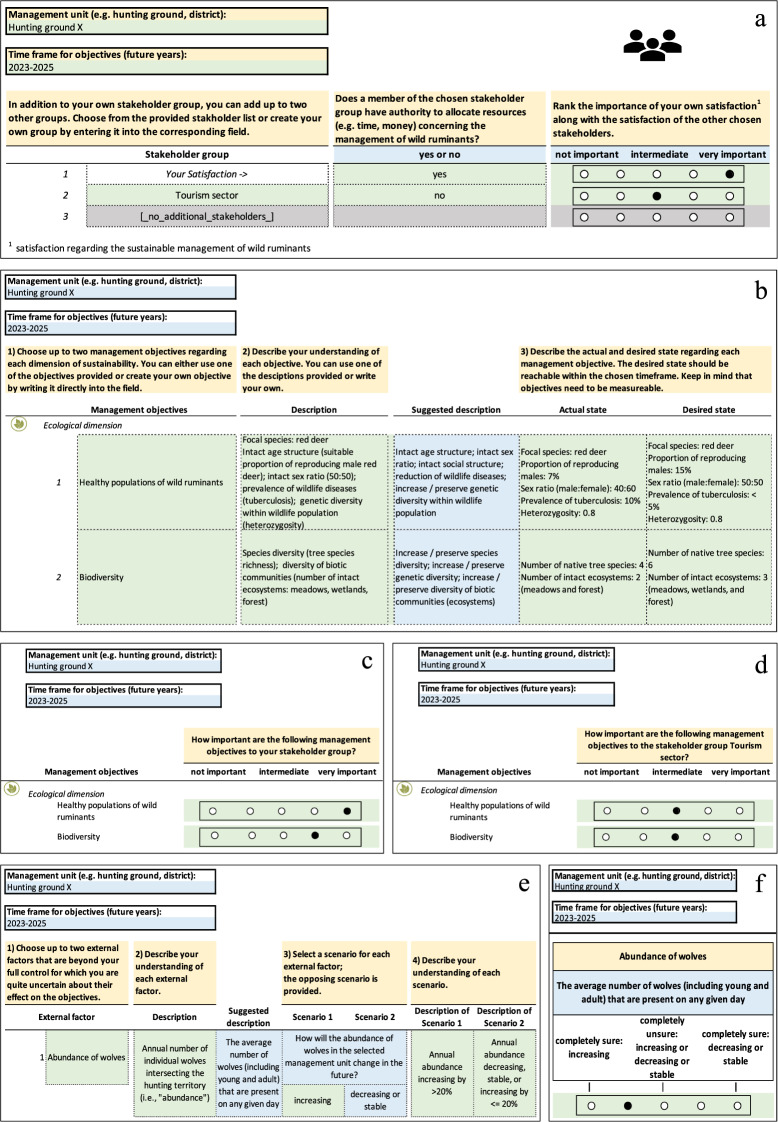
**a** Hypothetical example illustrating elicitation of the management unit, time frame, and stakeholders within the JAKEtool. **b** Hypothetical example illustrating elicitation of objectives within the JAKEtool. For simplicity, a subset of the objectives is shown. **c**, **d** Importance of objectives are entered separately for each stakeholder group along a 5-point scale. **e** Hypothetical example illustrating elicitation of an external factor within the JAKEtool. Up to two may be specified; one is shown for simplicity.** f** Level of uncertainty about future trajectory is entered for each external factor along a 5-point scale. Yellow fields: fields providing general instructions, green fields: fields to edit, blue fields: fields providing information regarding the choices available, gray fields: no data entered

The core team identified six stakeholder groups that affect or are affected by the management of wild ruminants in Lower Austria: (1) the forestry sector, (2) the agricultural sector, (3) the hunting sector, (4) the tourism sector, (5) the nature conservation sector, and (6) private landowners. Within the first two workshops, an individual representing each of these groups described their concerns regarding the management of wild ruminants in Lower Austria. Based on moderated group discussions, the following decision question was formed: “How can sustainability of management of wild ruminants in Lower Austria be maximized, under consideration of all affected stakeholder interests within a certain spatial extent?”. Stakeholders or decision makers involved with managing wild ruminants might be focal user of our tool. As a first step in the tool, the focal user identifies the management unit and time frame along with any relevant stakeholders, including decision makers, and scores their importance relative to their own importance within the decision context (Fig. 2a).

### Factors

We identified 24 objectives for inclusion within the factor catalog in the decision-support tool. Of these, seven were ecological, seven were economic, and ten were sociocultural. The frontend allows the user to select up to two objectives for each sustainability dimension (Fig. 2b). A proposed definition is provided, and the user may adapt this to their own decision context. The user is then asked to define the current and desired status of each objective, which is important information when providing predictions in a later step. Next, the user rates each selected objective based on an importance weight using a 5-point scale (Fig. 2c, d).

Based on a moderate to high uncertainty regarding their influence on objectives, eight external factors were chosen for inclusion in the factor catalog. The user can choose up to two external factors and later account for these when predicting effects of the decision options on the objectives. For each external factor, the user chooses a pair of potential scenarios like 1) increase vs. stable or decrease; or 2) increase or stable vs. decrease (Fig. 2e). The user is then asked to indicate the level of uncertainty about the future trajectory of each external factor along a 5-point scale (Fig. 2f).

The user does not see the probability associated with their response, but this probability is used for the decision-analytic computations in the backend of the tool. Workshop participants explicitly requested that the probabilities are indirectly elicited and hidden in the backend computations, as direct elicitation or display of probabilities would add cognitive burden for users without improving the quality of inputs.

**Fig. 3:**
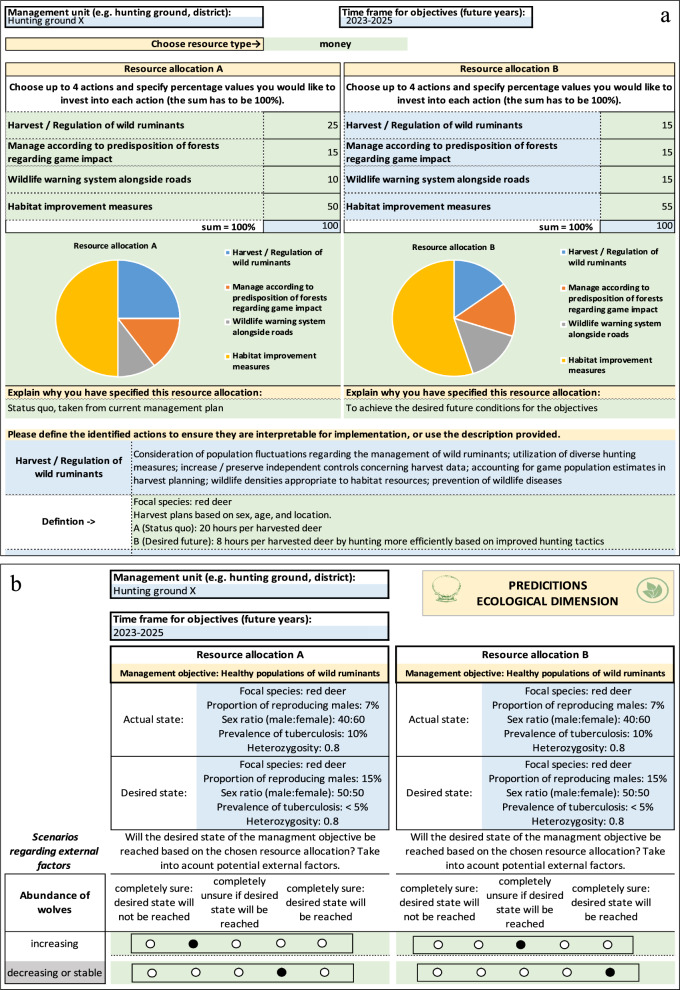
**a** Hypothetical example illustrating elicitation of resource allocations within the JAKEtool. A definition for each action may be entered; only one shown for simplicity.** b** Hypothetical example illustrating elicitation of predicted outcomes for the first ecological objective within the JAKEtool. Yellow fields: fields providing general instructions, green fields: fields to edit, blue fields: fields providing information regarding the choices available

We identified 21 possible actions that could be implemented to achieve one or more objectives. As some of these actions could affect objectives falling under multiple dimensions of sustainability, the actions were not subdivided among the dimensions. The user then constructs decision options as two possible ways of allocating resources among actions. In particular, the user indicates which type of resource will be allocated (i.e., time or money), they then select two to four actions, and finally they allocate the resources among the actions (Fig. 3a). The tool later compares the performance of these two resource allocations and visualizes the allocations as pie charts. As an alternative, we considered having the user simply choose two contrasting sets of actions for each decision option. Workshop participants preferred giving the allocation percentages, as this provides a more concise and transparent description of each decision option.

### Consequences, trade-offs, and backend computations

**Fig. 4:**
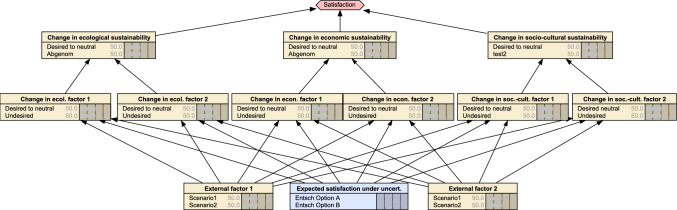
Bayesian decision network (BDN) developed using program Netica, which was used to verify results provided by the JAKEtool. Yellow boxes: stochastic nodes, blue box: decision node

A Bayesian decision network (BDN) was deemed suitable for computing expected performance of each decision option in the backend of the tool. In particular, the BDN allows for comparing decision options while accounting for their uncertain effects on one or more objectives (Mattsson et al. 2019). This comparison takes into account not only uncertainties about effects of decision options on objectives but also the compounding effects of external factors. The facilitators constructed the BDN with two decision options in a decision node and 11 stochastic nodes using program Netica (Fig. 4, Appendix S3). The stochastic nodes include two external factors, three ultimate objectives representing the dimensions of sustainability, and two objectives for each dimension. Each external factor node has two states represented by the two scenarios. Likewise, each objective node has two states that, respectively, represent achieving and failing to achieve the desired condition for that objective. Finally, each dimension of sustainability has two states representing possible trajectories in the future: “increase or stable” vs. “decrease”.

The user provides the predicted outcome under each possible combination of decision option and trajectory for the external factors along a 5-point scale (Fig. 3b), which is analogous to the approach used for predicting the trajectory of each external factor. The scale represents the range of uncertainty and the direction of the effect. The participants were adamant that the predictions be simplified to this extent to ensure that non-technical users could readily fill out the tool. This simplification, however, allowed us to convert the user input into a conditional probability for the corresponding node in the BDN. The calculations for the BDN were then implemented in the backend of the tool and verified by comparing results after entering trial values in the conditional probability tables for the stochastic nodes and percent satisfaction values in the utility node.

The second type of input required for the BDN is the utility (i.e., level of satisfaction) associated with each possible combination of objective states. We tried directly eliciting these utilities from workshop participants, and they were concerned that this placed a large cognitive burden. In short, they questioned the ability of users to directly and accurately quantify their satisfaction in this way. Instead, based on further discussion with the participants, the facilitation team designed the tool for deriving the utilities and corresponding trade-offs between objectives based on an importance weight for each objective elicited using a 5-point scale (Fig. 2c, d). The backend of the tool converts this entry into a score as follows: 1 point = 0% importance, 2 points = 25% importance, 3 points = 50% importance, 4 points = 75% importance, and 5 points = 100% importance. The importance weights for the individual objectives were then used to compute utility values for all possible combinations of states for the three sustainability dimensions.

Computing utilities assumed an additive utility function (Clemen and Reilly 2013) with independence among sustainability dimensions and comprised four steps. First, the importance score for each sustainability dimension was determined based on the average importance score between the associated objectives. Second, based on these importance scores, all possible combinations of states were ordered from 1 to 8 with the best possible state having a rank of 1 and the worst possible outcome having a rank of 8. Third, the top rank (1) was assigned a utility of 100 and the lowest rank (8) was assigned a utility of 0.

**Fig. 5:**
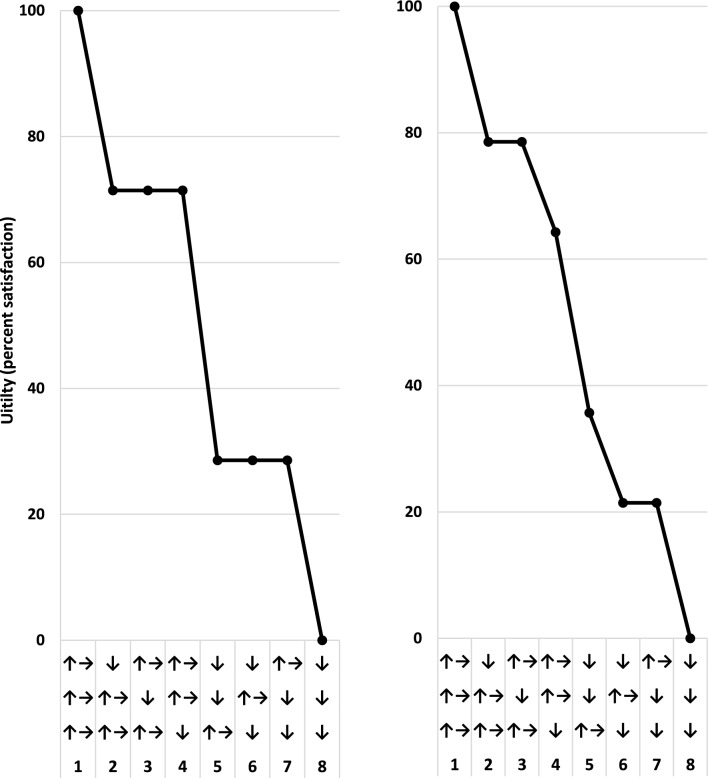
Utility. Modeled levels of satisfaction (i.e., utility) among eight combinations of states regarding changes in an index representing each of the three dimensions of sustainability. The index is the average of importance scores between objectives under a given dimension. The index and scores are not graphed, but changes in the indices are represented by arrows: ↑→ = increase or stable; ↓ decrease. Satisfaction regarding intermediate scenarios (i.e., states 2 through 7) depend on the ranking of importance scores among the dimensions by users of a decision-support tool as illustrated by two examples: (left) three-way tie among dimensions; (right) tie for second rank between ecological and economic dimensions

In the fourth and final step, these ranks and corresponding utility values were fit to a linear regression (Fig. 5):

$$U = 114.29 - 14.286 \times r$$

where *U* is the utility value, and *r* is the rank of a focal state. When scores were equal between dimensions of sustainability, the average rank was computed among tying members. If all scores were tied, then the rank was set to 3 for all combinations that had one decreasing state (i.e., state numbers 2–4). Continuing with this example, the rank was set to 6 for all combinations that had two decreasing states (i.e., state numbers 7–8). If scores were equal between two of the three dimensions, then the rank was set to 1.5 for tying states 1 and 2 (i.e., one of the focal states decreasing) and to 6.5 for tying states 6 and 7 (i.e., both focal states decreasing). The rank for each non-tying state was altered by 0.5 to maintain the assumption of an additive utility function. In particular, state 4 was given a rank of 3.5 and state 5 was given a rank of 5.5.

The JAKEtool then compares performance of two decision options using an expected utility (i.e., expected satisfaction), which is computed using the BDN algorithm:

$$E\left( {U_k } \right) = \sum \limits_i \sum \limits_j \left( { \sum \limits_a \sum \limits_b \left( {\Pr (\dim_{ijkab} ) \times \Pr ({\text{extfactor}}_{ab} )} \right) \times U_{ij} } \right)$$

where *i*, *j*, and *k* index the three sustainability dimensions, two sustainability dimension states (dim), and the two decision options, respectively. The external factors and external factor states (extfactor) are, respectively, indexed by *a* and *b*. The first term is the probability (Pr) of a sustainability dimension state j for dimension *i* given a decision option *k* and external factor state *b* of external factor *a*. The probability of the latter is represented by the second term. The probability of each sustainability dimension state is given as:

$$\Pr (\dim_{ijkab} ) = \frac{1}{n}\mathop \sum \limits_r^n \Pr ({\text{obj}}_{ijkabr} )$$

where *r* indexes the *n* objectives (obj) corresponding to sustainability dimension *i*. State *j* of the sustainability dimension *i* is set equal to that of the corresponding objective. For example, if the focal dimension state is optimistic (i.e., stable or increasing), then the corresponding state *r* for each objective within that dimension is set to achieve the desired condition for each objective. By calculating the average between probabilities of the objectives under sustainability dimension *j*, we assume that the *n* objectives have identical influence on the trajectory of this dimension.

**Fig. 6:**
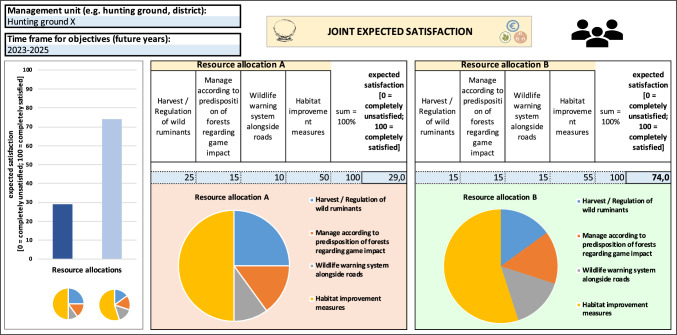
Hypothetical example illustrating the comparison between expected satisfaction averaged across stakeholder groups within the JAKEtool. An analogous result is provided for the focal user, but for simplicity this is not shown here. Yellow fields: fields providing general instructions, blue fields: fields providing information regarding the choices available

The final result of the tool consists of two graphs. The first graph compares the expected satisfaction of the focal user with respect to the two decision options. The second graph makes the same comparison but regarding the expected satisfaction averaged across all stakeholder groups, including that of the focal user (Fig. 6).

### Prospects for usefulness of the tool

Of the seven workshop participants who responded to the survey administered between the third and fourth workshop, five were quite satisfied (score = 4 of 5) and two were neither satisfied nor dissatisfied (score = 3 of 5) with the management of wild ruminants in Lower Austria. Therefore, none of the participants said they were extremely satisfied, revealing the opportunity to improve or to maintain their current level of satisfaction through the support provided by the tool. Based on results from the survey during the fifth workshop, five workshop participants were quite satisfied, and one was neither satisfied nor dissatisfied with the presented version of the tool. This version was largely identical to the final one, aside from minor adjustments. The deputy director of the forestry agency in Lower Austria, the main funder of the project, verbalized positive statements about the tool during the workshops. For example, he asserted that the tool has strong potential to inform real-world decisions regarding management of wild ungulates while raising awareness among practitioners about the complexity of such decision-making. Through the use of the tool, he expects that dynamics within this complex system will become more understandable and explicitly taken into account in decision-making processes.

### Tool demonstration

We filled out the tool for a hypothetical hunting territory in Lower Austria based on our knowledge and experience (Appendix S4). In particular, we identified the stakeholders, objectives, external factors, and actions to represent a realistic case. The intent was to illustrate the practical application of the tool while preparing a worked example that did not reveal private information from actual decision makers.

## Discussion

We built a ready-to-use decision-support tool for decision makers and stakeholders of various backgrounds faced with decisions within the management of wild ruminants at a fine spatial scale. Although developed based on decision contexts in Lower Austria, we believe it can be readily adapted for use in any region around the world. It allows for gaining a conceptual understanding of a complex decision along with a quantitative comparison of user-selected management options. The iterative nature of the tool development and providing written protocols of the workshops helped to build trust in the process while tailoring modifications of the decision model to the interests and needs of decision makers and stakeholders. Although we were unable to test real-world applications of the tool due to time constraints, all participants, except for one, indicated that the tool would be effective at addressing obstacles to their decision-making.

Although the decision tool represents an important advance for sustainable management of wild ruminants, we recognize areas for future work to increase its robustness and usefulness. The most important need is to develop the tool as an open-source web application that is fast and user friendly. The Excel macros are often slow and navigating between the sheets can be cumbersome. These difficulties may discourage some practitioners from using the existing tool. Due to insufficient resources available, we were not able to develop an open-source web application or implement a stand-alone graphical user interface. When necessary resources become available, an important next step is to develop such a web application for broader use.

Predictive models did not exist for parameterizing predictions in the decision model. The tool therefore relies on users to specify levels of uncertainty regarding effects of external factors and resource allocations on objectives. The decision model structure is suited for calculating expected value of perfect information (Runge 2011), which would quantify the level of importance for reducing each uncertainty. Practitioners could then work with researchers if needed to address these uncertainties through literature review, data collection, and predictive modeling. Identifying uncertainties to address through linking management and monitoring could form the basis for a formal adaptive management program that includes stakeholder involvement (Williams and Brown 2014; Mattsson et al. 2018). The tool could be extended to update model weights based on information collected by practitioners or researchers, and these weights would in turn improve model predictions and increase likelihood of achieving objectives (e.g., Nichols et al. 2007; Powell et al. 2022).

The participants in our study were satisfied with the simple design of the tool, but there may be users who would like to explicitly address more complex decision contexts or access advanced features. To accommodate additional complexity, the BDN could be administered using code. Implementing the decision model in a programming language would allow for more efficient generation of additional nodes and edges representing added factors and corresponding relationships in the decision model. The code would be embedded in the backend of the web application mentioned above to maintain the accessibility of the tool for practitioners.

An advanced feature that would increase the robustness of results from the tool would relax the assumption of an additive utility function. An open-source web application could include an interactive graph that allows the user to adjust their utility function after eliciting importance scores for the objectives (Dewancker et al. 2016). This would minimize elicitation burden while allowing for a more rigorous analysis tailored to the more nuanced trade-offs faced by practitioners.

Another important need is to evaluate the usefulness of the tool in real-world applications. This should be conducted using a before-after-control-impact design to obtain robust inferences (Eikelboom and Janssen 2017). Furthermore, hunting collectives are randomly selected to ensure a representative sample within a given federal state or region. Before and after applying the tool to a focal decision, researchers would survey practitioners about their satisfaction with the management of wild ruminants in the selected hunting collective. Statistical analyses would examine the potential difference in satisfaction before and after the use of the tool in control (i.e., nonuser of tool) and treatment (i.e., user of tool) groups. Finally, qualitative interview questions regarding the usefulness of the tool can be applied for evaluation and making improvements. These findings would allow for a better understanding of not only the immediate usefulness of the tool but also how it could be further improved.

## Conclusion

Worldwide, modern wildlife management is faced with complex problems. As stakeholder interests are diverse and oftentimes contradicting, decision tools offer great possibilities in applying a theory of change (Allen et al. 2017) in addressing complex situations and therefore mitigating wildlife-based conflicts. Hence, such tools can be helpful additions to the wildlife management toolbox in many countries. Structuring objectives according to the pillars of sustainability while allowing users to select particular objectives, actions, and external factors would ensure that the results are applicable to real-world decisions. Otherwise, decision tools are likely to fail in practical usability and be only useful in an academic context. We furthermore strongly argue for integrative and participatory approaches such as stakeholder workshops to inform development of decision tools. Engaging with stakeholders of all perspectives within complex problems is a key principle of sustainability sciences (van Kerkhoff 2014). During the stakeholder workshops, we elicited practical information and developed our tool in accordance with mission-driven science (International Science Council 2021).

Our tool is responsive to real-world needs of game managers around the globe, as users can define the decision context for applying the tool. Further, the tool is supportive to policy and practitioners in that quantitative comparisons of management options provide a level of transparency that is often sought by natural resource managers (International Science Council 2021). We thus enhanced the research-action interface for sustainability (van Kerkhoff and Lebel 2006) by integrating decision science in a decision-support tool designed for practitioners concerned about ecological, economic, and sociocultural aspects in the systems they manage. Although the tool was tailored for management of wild ruminants, the underlying structure is applicable to other sectors of natural resource management dealing with sustainability issues including forestry, agriculture, nature-based tourism, fisheries, nature conservation, and regional planning. Thus, our tool represents an important step toward developing and evaluating a transparent and replicable approach for mitigating wildlife-based conflicts worldwide.

### Supplementary Information

Below is the link to the electronic supplementary material.

Supplementary file1 (PDF 224 kb)
