# Supplementary material for: Building a decision-support tool to inform sustainability approaches under complexity: Case study on managing wild ruminants
Source: Ambio. 2024 Apr 17;53(9):1307–22. doi: 10.1007/s13280-024-02020-9 (PMC11300410; doi:10.1007/s13280-024-02020-9)
Supplement: Supplementary file 1 — Supplementary file1 (PDF 224 kb) [file 13280_2024_2020_MOESM1_ESM.pdf]

Ambio

Supplementary Information

This supplementary information has not been peer reviewed.

Title: **Building a decision-support tool to inform sustainability approaches under complexity: case study on managing wild ruminants**

Appendix S1

Questions during the first workshop

Q1.1 [multiple choice]: Please choose your level of satisfaction concerning how the annual game harvest plan is currently prepared and how it is fulfilled.

Q1.2 [text field]: Please briefly state the reason for your answer.

Questions after the first and before the second workshop

Q2.1 [text field]: Is the current form of hunting management on wild ruminants in Lower Austria sustainable and expedient? If NO, what do you suggest should be improved?

Q2.2 [text field]: Please describe the most needed action to be implemented to increase the level of sustainability within the game management of wild ruminants. Also note actions already in place that you think are of high importance to increase sustainability within the game management of wild ruminants.

Q2.3 [text field]: Who (persons, authorities, organizations) would be responsible to implement the aforementioned actions?

Q2.4 [text field]: What fundamentals are needed to implement those (new) actions?

Q2.5 [text field]: Are those actions meant to be implemented once or as long-term plans, maybe periodically?

Q2.6 [text field]: Assuming your suggested actions will be implemented accordingly, what impact/outcome do you expect or wish for?

Q2.7 [text field]: What are external factors that are not part of your actions but will have an effect on the actions or their results?

Q2.8 [text field]: Do you have any chance to affect these external factors? If so, what would that be and how would it affect the external factors?

Q2.9 [text field]: Should the goal of the project (*Enabling self-evaluation of game management on wild ruminants in regards to sustainability*) be adapted or changed based on what you feel would be more important?

Q2.10 [text field]: Are there any ideas or suggestions you want to make concerning the upcoming second workshop?

Questions after the second and before the third workshop

Q3.1 [text field]: Please define “sustainability”.

Q3.2 [text field]: Please describe your understanding of sustainable game management of wild ruminants.

Q3.3 [multiple choice]: Please check each management strategy/option which you can influence/use/prompt. [For options see Actions in Annex Catalog]

Q3.4 [text field]: Concerning your position, are you authorized to issue these strategies/options in Lower Austria?

Q3.5 [text field]: At which spatial level are you authorized to issue one or more strategies/options?

Q3.6 – 3.13 [text field]: If you issue one or more strategies/options, are those over the whole district/hunting collective/municipality/hunting territory, in all to them, or only in subareas?

Q3.14 [integer between 0 and 100 for each option]: Which percentage of your available resources (time and money) would you be willing to invest into these strategies/options?

Q3.15 [text field]: Please note the time frames you would be willing to prompt these strategies/options.

Q3.16 [text field]: Please describe your power and influence in implementation of these strategies/options.

Q3.17 [text field]: Do you see any problems or challenges in conjunction with these strategies/options? If so, please describe them briefly.

Questions during the third workshop

Q3.18 [multiple choice]: Are you satisfied with the decision-support tool as it is right now?

Q3.19 [text field]: Please briefly state the reason for your answer.

Q3.20 [text field]: Are there possible applications of the decision-support tool as it is now or of any improved version of it? If so, please describe them briefly.

Q3.21 [text field]: Are there problems or challenges concerning the usability of the decision-support tool? If so, please describe them briefly.

Q3.22 [text field]: Would you be willing to spend more time using the decision-support tool if it would offer more possibilities and therefore increase its capability of providing better support? If yes, how much more time would you be willing to spend?

Questions after the third and before the fourth workshop

Q4.1 [text field]: Please define "sustainability".

Q4.2 [text field]: Please describe your understanding of sustainable game management of wild ruminants.

Q4.3 [multiple choice]: Please choose your level of satisfaction concerning how the annual game management of wild ruminants is organized in Lower Austria

Q4.4 [text field]: Please elaborate the reasons for your choice in Q4.3.

Q4.5 [text field]: At which spatial level (e.g., district/hunting collective/municipality/hunting territory) are you acting within the game management of wild ruminants?

Q4.6 [multiple choice]: Please choose your level of satisfaction concerning how the annual game management of wild ruminants is organized at your specific spatial level.

Q4.7 [text field]: Please elaborate the reasons for your choice in Q4.6.

Q4.8 [multiple choice]: Please check each management strategy/option for which you can decide on how resources (money, time) will be used. [For options see Actions in Annex Catalog]

Q4.9 [single choice]: Are you empowered to issue these strategies/actions mentioned in Q4.8 within the whole province of Lower Austria? [Yes/No]

Q4.10 [text field]: At which spatial level (e.g. district/hunting collective/municipality/hunting territory) are you authorized to issue one or more strategies/options?

Q4.11 – 4.18 [text field]: These questions asked whether the participant is able to act on the whole area of his/her spatial level or only part of it. Two questions per spatial level: district, hunting, collective, municipality, hunting territory

Q4.19 [text field]: For what period of time would you issue strategies/options?

Q4.20 [text field]: Please describe your power and influence in implementation of the strategies/options.

Q4.21 [text field]: Do you see any problems or challenges in conjunction with the strategies/options? If so, please describe them briefly.

Questions during the fourth workshop

Q4.22 [multiple choice]: Are you satisfied with the decision-support tool as it is right now?

Q4.23 [text field]: Please briefly state the reason for your answer.

Q4.24 [text field]: Are there possible applications of the decision-support tool as it is now or of any improved version of it? If so, please describe them briefly.

Q4.25 [text field]: Are there problems or challenges concerning the usability of the decision-support tool? If so, please describe them briefly.

Q4.26 [text field]: Would you be willing to spend more time using the decision-support tool if it would offer more possibilities and therefore increase its capability of providing better support? If yes, how much more time would you be willing to spend?

Questions during the fifth workshop, first of two questionnaires

Q5.1 [text field]: Please note one administration unit for which you could issue strategies/options and decide on how resources (money, time) will be used.

Q5.2 [text field]: For what period of time would you issue strategies/options?

Q5.3 [text field]: Would you like to include other stakeholders within your decision analysis? If so, which ones?

Questions during the fifth workshop, second of two questionnaires

Q5.4 [multiple choice]: Are you satisfied with the decision-support tool as it is right now?

Q5.5 [text field]: Please briefly state the reason for your answer.

Q5.6 [text field]: Is there potential to improve the decision-support tool as it is right now? If so, please clearly state the exact stage.

Q5.7 [text field]: Do you like to participate in the last workshop and see the final decision-support tool?

Questions after the fifth and before the sixth workshop

Q6.1 [multiple choice]: How likely are you employing the decision-analysis tool within your area to inform decisions on management of wild ruminants?

Q6.2 [text field]: Are there necessary changes for the decision-analysis tool to be used? If so, which ones?

Q6.3 [multiple choice]: How likely are you employing the decision-analysis tool within your area to inform decisions on management of wild ruminants AFTER changes mentioned in Q6.2 would be employed?

Q6.4 [text field]: Please briefly state the reason for your answer in Q6.3.

Q6.5 [text field]: What would be necessary to increase the probability of you using the decision-analysis tool?

Q6.6 [multiple choice]: Would you need support by a trained moderator when using the decision-analysis tool? [Yes/No/Maybe]

Q6.7 [multiple choice]: Technical context: How would you use the decision-analysis tool? [Desktop/Web-based/Smartphone-based/Hardcopy print-outs/Else]

Q6.8 [multiple choice]: Social context: How would you use the decision-analysis tool? [Alone or with moderator/during meeting with colleagues/during meeting with stakeholders/Else]

Q6.9 [text field]: Do you have any further ideas where the decision-analysis tool could be used?

Appendix S2

Developed decision-support tool (Download):

https://drive.boku.ac.at/f/e6625493034a4516ad65/?dl=1

Note: Macros have to be activated for the tool to work. Due to functions used, Excel version 2021 or higher is required for the tool to work properly.

Appendix S3

Bayesian decision network with two decision options in a decision node and 11 stochastic nodes using program Netica (Download):

https://drive.boku.ac.at/d/79c7bf2af84f47cbba8e/files/?p=%2FBDN%20JAKE%20en%20example%20ms.neta

Note: To open the file, Netica Application Software has to be installed.

Appendix S4

Tool demonstration for a hypothetical hunting territory in Lower Austria (Download):

https://drive.boku.ac.at/f/5066fbe57ba1469887a3/?dl=1

Note: Macros have to be activated for the tool to work. Due to functions used, Excel version 2021 or higher is required for the tool to work properly.

Table S1 Catalog of factors (i.e., objectives, actions, and external factors) for selection by users of a decision-support tool to inform sustainable hunting of wild ruminants. The spatial extent for each factor is the selected management unit, and the time frame over which factors are projected is specified elsewhere by the user. Descriptions are provided in the tool as initial suggestions that the user can adapt as needed. Care must be taken when refining the definitions of factors to ensure that they retain their character (e.g., objective remains an objective rather than an action) and are mutually exclusive.

Ecological dimension

No.	Management objective	Definition
1	**Healthy populations of wild ruminants**	Intact age structure; intact sex ratio; intact social structure; reduction of wildlife diseases; increase / preserve genetic diversity within wildlife population
2	**Habitats suitable for wild ruminants**	Creation of wildlife corridors (linking fragmented ecosystems or favorable habitats); consideration of wildlife habitat requirements (habitat quality: climate, food, shelter, disturbance, etc.); consideration of spatial and temporal habitat change; consideration of habitat resources
3	**Forestry objectives regarding the ecological dimension of sustainability**	Securing ecological forest functions; increase / preserve number of site-appropriate tree species; forests adapted to climatic changes; preserve forests as habitats for wildlife
4	**Wildlife densities appropriate to habitat resources**	Individuals per species per hectare
5	**Biodiversity**	Increase / preserve species diversity; increase / preserve genetic diversity; increase / preserve diversity of biotic communities (ecosystems)
6	**Spatiotemporal space use of wild ruminants**	Maintain or restore natural grazing rhythm of wild ruminants
7	**Ecological objectives across neighboring hunting grounds**	Increase / maintain one or more ecological objectives (e.g., from the list above) in the focal hunting ground and adjacent ones

Economic dimension

No.	Management objective	Definition
8	**Damages caused by wild ruminants on forests or agricultural land**	Prevention of unacceptable game impacts (bark stripping, browsing, etc.) on forest and agricultural land; reduction of damage on forest vegetation through browsing from … € to … €
9	**Economic value of hunting**	Increase / preserve hunting tourism; leasing of hunting grounds; increase / preserve hunting profitability; increase of hunting revenues
10	**Road safety regarding the economic dimension of sustainability**	Reduction of road kills; decrease of car accidents caused by wild ruminants
11	**Forestry objectives regarding the economic dimension of sustainability**	Increase / preserve economic forest functions (e.g. increasing the profit through silvicultural use of a forest)
12	**Profits earned by hunters and stakeholders through hunting practices**	Increase net earnings by hunters, forest managers, farmers, recreationists and further stakeholders through the practice of game management.

13	**Efficient hunting strategies to achieve economic objectives**	Reduction of resources (time and money) needed to harvest wild ruminants
14	**Economic objectives across neighboring hunting grounds**	Increase / maintain one or more economic objectives (e.g., from the list above) in the focal hunting ground and adjacent ones

Socio-cultural dimension

No.	Management objective	Definition
15	**Hunting traditions and customs**	Increase / preserve traditions and customs regarding hunting (e.g. dress code, behavior towards game species, music)
16	**Image and status of hunting in society**	Increase / preserve the image of hunting in society
17	**Road safety regarding the socio-cultural dimension of sustainability**	Prevention of injured persons
18	**Balancing of various interests**	Decrease of conflicts between stakeholders
19	**Hunting ethics**	Maintain compliance with hunting code of ethics
20	**Compliance with legal framework conditions**	Decrease number of violations regarding the hunting law and nature conservation law
21	**Freedom of landowners with regard to decision-making**	Minimizing intervention by government authorities in decision-making; Maximize ability to incorporate objectives that are tailored for a particular socio-cultural context
22	**Hunting recreational value**	Preserve hunting as a recreational activity with responsibility
23	**Forestry objectives regarding the socio-cultural dimension of sustainability**	Increase / preserve socio-cultural forest functions (e.g. forests as recreational areas)
24	**Awareness about impacts between hunters and stakeholders**	Increase knowledge about effects of hunting on other sectors and vice-versa

Actions

No.	Action	Definition
1	**Education for hunters**	Improve or update knowledge in the field of wild ruminant biology; administer lectures/seminars regarding effects of hunting on other sectors and vice-versa; raise awareness of the influence of hunters on the image of hunting in society; preservation of hunting traditions and customs
2	**Integral coordination**	Increase / preserve cooperation with researchers (e.g., wild ruminant biology); increase / preserve coordination of actions with other stakeholders to reduce game impact; increase / preserve cooperation with neighboring hunting grounds to be able to define decision options
3	**Habitat improvement measures**	Create wildlife corridors; establish grazing areas; create suitable shelter (areas that provide protection from weather and predators) for wild ruminants; consider wildlife ecology aspects (e.g. suitable habitats) in land use

4	**Supplementary feeding of wild ruminants**	Increase / preserve grounds in providing supplemental food; increase / preserve cooperation with neighboring hunting grounds; compensate for habitat loss with winter feeding; prevention of game impact (e.g., bark stripping, browsing)
5	**Hunting concepts across hunting grounds**	Increase / preserve wildlife ecological spatial planning (Reimoser 1999); establish efficient hunting concepts to ensure forest functions regarding all dimensions of sustainability
6	**Integrate aspects of wild ruminant biology in management decisions**	Adaptations of management decisions to the biology of wild ruminants like reproductive biology or life-cycle
7	**Anthropogenic selection related to the harvest of wild ruminants**	Reduce selective hunting of wild ruminants with certain traits (e.g. antler sizes)
8	**Healthy populations of wild ruminants**	Increase / preserve healthy populations of wild ruminants; harvest of sick and weak animals
9	**Availability of wild ruminants for harvest**	Increase / preserve availability of wild ruminants by adjustments of hunting pressure; increase / preserve wildlife ecological spatial planning (Reimoser 1999); increase / preserve integral coordination between diverse stakeholders; alterations of anthropogenic predation risk; increase / preserve combinations of diverse hunting strategies
10	**Harvest / Regulation of wild ruminants**	Consideration of population fluctuations regarding the management of wild ruminants; utilization of diverse hunting measures; increase / preserve independent controls concerning harvest data; accounting for game population estimates in harvest planning; wildlife densities appropriate to habitat resources; prevention of wildlife diseases
11	**Spatio-temporal distribution of wild ruminants**	Altering the spatio-temporal habitat use by wild ruminants by creating diverse anthropogenic predation risks across the landscape to reduce game impact on forest or agricultural land
12	**Wildlife warning system alongside roads**	Reduction of road kill; increase of road safety to counteract damages to cars or humans; increase / preserve the utilization of optical and acoustic wildlife warning systems alongside roads
13	**Hygienic aspects regarding harvested game**	Specify and increase / preserve the control regarding the handling of harvested game from the killing of the animal to the marketing of the meat
14	**Manage according to predisposition of forests regarding game impact**	Increase / preserve the consideration of different forest types and their predisposition regarding game impact on forests when managing wild ruminants
15	**Hunting infrastructure**	Increase / preserve the number of hides; increase / preserve the number of shooting lanes; increase / preserve hunting infrastructure as preparation for leasing (preservation of the economic value of hunting); taking care of hunting equipment
16	**Marketing strategy regarding hunting**	Using game as a marketing product; hunting as service provider for society by advertising approaches to regulating wild ruminants and reducing game impact on forest or agricultural land
17	**Legal control**	Verify compliance with law by cooperation with control organs
18	**Fundamental scientific research**	Increase / preserve investments regarding fundamental research on wild ruminants to better understand wildlife biology; increase / preserve cooperation with science
19	**Awareness regarding the positive effects of hunting**	Increase / preserve awareness regarding the positive effects of hunting via education campaigns, posting signs, social-media, etc.
20	**Education for recreationists who are non-hunters**	Increase / preserve awareness of non-hunting recreationists regarding human impacts on wildlife

External factors

No.	External factor	Definition
1	**Abundance of wolves**	The average number of wolves (including young and adult) that are present on any given day
2	**Use of the landscape by recreationists**	The intensity of landscape use by recreationists (e.g., average number of recreationists per day)
3	**Precision and accuracy in assessment of game impact**	The amount and quality of measurements to determine the impact of wild ruminants on forest condition (e.g., Nopp-Mayr et al. 2020)
4	**Predisposition of forests regarding game impact**	The vulnerability of forests to game impacts associated with environmental conditions (e.g., soil and climatic conditions) and human influences besides game management (e.g., forest management; e.g., Nopp-Mayr et al. 2011)
5	**Hunting suitability**	Suitability for hunting as it pertains to environmental conditions (e.g., landscape composition and forest structure) and human influences besides game management (e.g., forest management) (e.g., Griesberger et al. 2022)
6	**Forest habitat condition**	Status of the forest habitat condition (regarding wild ruminants) as it relates to silvicultural practices (e.g., Vospernik & Reimoser, 2008)
7	**Influence of climate change on forests**	The influence of climate change on tree species composition

Griesberger, P., L. Obermair, J. Zandl, G. Stalder, W. Arnold, and K. Hackländer. 2022. Hunting suitability model: a new tool for managing wild ungulates. *Wildlife biology* May 2022: 1–11. doi:10.1002/wlb3.01021.

Nopp-Mayr, U., F. Reimoser, and F. Völk. 2011. Predisposition assessment of mountainous forests to bark peeling by red deer (Cervus elaphus l.) as a strategy in preventive forest habitat management. *Wildlife Biology in Practice* 7: 66–89. doi:10.2461/wbp.2011.7.7.

Nopp-Mayr, U., S. Reimoser, F. Reimoser, F. Sachser, L. Obermair, and G. Gratzer. 2020. Analyzing long-term impacts of ungulate herbivory on forest-recruitment dynamics at community and species level contrasting tree densities versus maximum heights. *Scientific Reports* 10. Nature Publishing Group UK: 1–13. doi:10.1038/s41598-020-76843-3.

Vospernik, S., and S. Reimoser. 2008. Modelling changes in roe deer habitat in response to forest management. *Forest Ecology and Management* 255: 530–545. doi:10.1016/j.foreco.2007.09.036.
